# Bevacizumab and radiotherapy for the treatment of glioblastoma: brothers in arms or unholy alliance?

**DOI:** 10.18632/oncotarget.6320

**Published:** 2015-11-13

**Authors:** Maximilian Niyazi, Patrick N. Harter, Elke Hattingen, Maya Rottler, Louisa von Baumgarten, Martin Proescholdt, Claus Belka, Kirsten Lauber, Michel Mittelbronn

**Affiliations:** ^1^ Department of Radiation Oncology, University of Munich, Germany; ^2^ German Cancer Consortium (DKTK) and German Cancer Research Center (DKFZ), Heidelberg, Germany; ^3^ Institute of Neurology (Edinger Institute), Goethe University, Frankfurt, Germany; ^4^ Department of Neuroradiology, University Hospital Bonn, Bonn, Germany; ^5^ Department of Neurology, University of Munich, Munich, Germany; ^6^ Department of Neurosurgery, University Hospital Regensburg, Regensburg, Germany

**Keywords:** glioma, radiotherapy, bevacizumab, angiogenesis, VEGF

## Abstract

Glioblastoma (GBM) represents the most frequent primary brain tumor in adults and carries a dismal prognosis despite aggressive, multimodal treatment regimens involving maximal resection, radiochemotherapy, and maintenance chemotherapy. Histologically, GBMs are characterized by a high degree of VEGF-mediated vascular proliferation. In consequence, new targeted anti-angiogenic therapies, such as the monoclonal anti-VEGF-A antibody bevacizumab, have proven effective in attenuating tumor (neo)angiogenesis and were shown to possess therapeutic activity in several phase II trials. However, the role of bevacizumab in the context of multimodal therapy approaches appears to be rather complex. This review will give insights into current concepts, limitations, and controversies regarding the molecular mechanisms and the clinical benefits of bevacizumab treatment in combination with radio(chemo)therapy - particularly in face of the results of recent phase III trials, which failed to demonstrate convincing improvements in overall survival (OS).

## BACKGROUND

GBMs are highly vascularized tumors that critically depend on the generation of tumor-associated blood vessels [1, 2]. They are characterized by a dense network of highly disorganized, tortuous, large-diameter vessels with increased basement membrane thickness [3, 4]. The pathological tumor vasculature is functionally abnormal and leads to increased vessel permeability, vasogenic edema and hemorrhages [5]. The resulting rich capillary network may contribute to rapid tumor growth and poor prognosis. As the vasculature in GBMs is characterized by a highly disorganized vessel architecture, this may also limit the efficacy of radio- and chemotherapy by compromising blood flow, thereby enhancing tumor hypoxia and impairing oxygen-mediated, irradiation-induced DNA damage as well as the delivery of chemotherapeutics [2, 6]. Among multiple factors controlling the complex process of angiogenesis, vascular endothelial growth factor (VEGF) and its associated signaling cascade are considered to be of central importance [5, 7]. Glioma cells are a major source of VEGF, and high levels of VEGF have been reported to correlate with high-grade malignancy and poor prognosis [8, 9]. Radiotherapy (RT) is a mainstay of GBM treatment and is known to dramatically increase VEGF expression by transactivating factors, including NFκB and p53 [10]. Accordingly, it was speculated that targeting VEGF might enhance the efficacy of RT. Upon inhibition of VEGF signaling the downstream effects (temporary increase in perfusion, decrease in interstitial edema/hypertension as well as permeability) should all support the efficacy of concomitant RT [5, 11].

Currently, the most prominent VEGF targeting drug is bevacizumab (BEV), a recombinant humanized monoclonal antibody that binds to human VEGF-A. Several groups have investigated the use of BEV with an already proven efficacy in metastatic colon, breast, and lung cancer for patients with recurrent malignant gliomas [12, 13]. BEV was also tested in combination with RT in recurrent GBM [14], or as upfront treatment together with RT and concomitant temozolomide (TMZ) administration [15, 16]. It proved to be a safe and feasible treatment option. The mode of action was attributed to scavenging mechanisms, which counteract irradiation-induced VEGF secretion by inactivating VEGF [17], thus resulting in consecutive advantageous effects, including reduction of vascular permeability and edema, improved oxygenation as well as reduction of radiation necrosis [5, 18]. Furthermore, several phase II trials have documented the efficacy of anti-VEGF therapies either alone or in combination with irinotecan [19-25], etoposide [26, 27], nitrosourea [28], or other agents [29, 30]. However, the initially reported promising response rates to BEV treatment might be at least in part - attributed to imaging limitations resulting from decelerated neoangiogenesis and reduced vascular permeability, thus leading to an apparent but debatable reduction in the contrast-enhancing tumor volume [31, 32].

Recent prospective phase III trials (AVAglio & RTOG 0825) were designed to prove the efficacy of TMZ-based radiochemotherapy in combination with BEV as first-line therapy for GBM. While the RTOG 0825 trial failed to show significant benefits in terms of progression-free (PFS) and overall survival (OS), the AVAglio study demonstrated a significant prolongation of PFS by 4.4 months in the BEV arm. However, this PFS benefit did not translate into an improvement in OS [33, 34]. In line with this, several preclinical and clinical studies raised the concern that BEV treatment might induce a more invasive tumor phenotype, thereby potentially limiting the efficacy of radiation therapy due to the stimulation of tumor cell emigration out of the RT field [35-38]. Moreover, an increased incidence of neurocognitive side effects was reported for the combination of RT and BEV treatment and is currently attributed to VEGF's role as neuroprotector [39]. Overall, due to the general limitations of the existing phase II/III trials, it remains controversial whether the initial preclinical rationale to combine anti-VEGF treatment and RT has to be regarded as corroborated or disproven [33, 34], and this is not the only important question in the field, which waits to be resolved. Here, we aim at providing an overview of the current knowledge, hypotheses, and discussions about the molecular mechanisms underlying the effects of BEV treatment in combination with irradiation and their clinical implications for the treatment of GBM patients.

## THERAPEUTIC EFFECTS OF BEV AND TREATMENT RESISTANCE IN PRECLINICAL GLIOMA MODELS

Anti-angiogenic therapy targeting VEGF or its receptors is considered to impact on glioma growth through several mechanisms, some of which are only poorly understood [40]. The initial hypothesis was that anti-angiogenic therapy prunes tumor vessels and reduces tumor blood perfusion, thereby starving the tumor of oxygen and essential nutrients, resulting in reduced tumor growth [41]. However, accumulating evidence indicates that one therapeutic effect of anti-VEGF treatment derives from transient normalization of the functionally abnormal tumor vasculature, leading to a reduction in edema and improved tumor oxygenation [42]. Preclinical studies suggest that this time window of normalization is important with regard to the schedule of combination therapies. Winkler et al. hypothesized that blockade of VEGF receptor 2 (VEGFR2) creates a “normalization window”. Within this time window, the combination with RT provides the best results of tumor control, originating from a temporary increase in tumor oxygenation, which is well known to enhance irradiation-induced DNA damage and thus irradiation-induced tumor cell death. Mechanistically, vascular normalization stimulated by VEGFR2 blockade emerges from increased pericyte coverage of brain tumor vessels via up-regulation of Angiopoietin 1 (Ang1) and degradation of the pathologically thick basement membrane via activation of matrix metalloproteases (MMPs) [43]. Dings et al. described similar findings for BEV treatment and VEGF inhibition [44]. Interestingly, a similar normalization effect has been described for radiotherapy itself or in combination with other treatment modalities [45, 46]. However, although the data on vascular normalization appear very promising, so far they remain limited to preclinical and extracerebral models.

In principle, the term “vascular normalization” might be misleading as some tortuous, large-diameter vessels will not immediately disappear upon administration of BEV (if not already removed by prior resection). But there is another reason, which supports the notion that RT and BEV treatment can act synergistically. The term “window of opportunity” as introduced by Jain et al. appears to describe the physiological effects that can be observed during RT and BEV treatment more precisely [5]. It does not seem to be an anatomically evaluable effect with less “disturbed” vessels, which turn “normal”, but rather a functional one. Normalization upon anti-VEGF treatment appears to be due to higher perfusion rates, less vascular permeability, less interstitial edema and hypertension, as well as less subsequent hypoxic burden. This, in turn, may immediately enhance the efficacy of RT and is from a kinetic point of view a short-term effect [11, 47]. Importantly, the potential adverse effects of long-term anti-VEGF treatment should not be confused with these obviously positive short-term effects as proven by preclinical trials [11, 43].

Apart from its impact on tumor vessel architecture and function, it remains controversial whether or not BEV possesses direct anti-tumor activity, and to date it is unknown if there is a threshold dose or dose-response relationship. We established an orthotopic mouse glioma model, which allowed us to simultaneously study the kinetics of morphological and functional vascular changes, tumor growth, and viability of individual tumor cells during the course of anti-VEGF therapy within the same microscopic tumor region in real-time. Notably, regression of gliomas occurred independently of vascular regression, suggesting that high doses of BEV have direct anti-cancer efficacy *in vivo* [48]. In line with this, a recent study provided evidence that gliomas expressing VEGFR2 comprise an aggressive subgroup of tumors, which develop resistance against TMZ and BEV treatment very early [49]. On the contrary, other preclinical studies could not reproduce the beneficial effects of anti-VEGF treatment on tumor growth control and improved survival in mice bearing orthotopically transplanted or autochthonous gliomas [50]. In this report, the described increase in progression-free survival times (PFS) in humans was interpreted as a decrease in vasogenic edema by a stabilization of tumor-associated brain microvessels and by a still controversially discussed increase in invasiveness, which in turn might potentially reduce the diagnosable local tumor mass, e.g. in eloquent CNS areas. Although VEGFR2 is traditionally regarded as an endothelial cell protein, there is accumulating evidence suggesting that VEGFRs may be expressed by cancer cells [49]. Hamerlik et al. proposed that VEGFR2 is preferentially expressed on the cell surface of CD133^+^ human glioma stem-like cells, whose viability, self-renewal, and tumorigenicity rely at least in part on signaling through the VEGF-VEGFR2-Neuropilin-1 (NRP1) axis. It was hypothesized that a limited impact of BEV-mediated VEGF blockage may reflect ongoing autocrine signaling through VEGF-VEGFR2-NRP1 [51]. If these findings can be directly transferred to human patients is highly questionable, since we and others could not corroborate the suitability of CD133 as a cancer stem cell marker in human gliomas [52].

## THE METABOLIC SWITCH INDUCED BY ANTI-ANGIOGENIC TREATMENT AND ITS RELEVANCE FOR THE EFFICACY OF RADIOTHERAPY

BEV treatment leads to a considerable change in the composition of metabolites in the CNS as assessed by neuroimaging. However, most aspects of the changes in the cellular and metabolic composition after anti-angiogenic treatment still remain to be defined. Meanwhile it is well acknowledged among physicians and scientists in the neurooncological field that BEV-treated gliomas reveal a more hypoxic, more glycolytic, and/or more invasive phenotype, although only limited experimental evidence supporting this issue is available [53]. This recycled statement mainly relies on cell culture or preclinical animal models. Detailed analyses were provided by Keunen and colleagues who characterized the effects of anti-VEGF treatment in intracranial glioblastoma xenografts [54]. In this study, a significant reduction in the cerebral blood flow and the amount of large and median-sized blood vessels upon anti-angiogenic treatment was associated with a dramatic increase in glioma cell invasion into the tumor-surrounding CNS. The tumor tissue became strongly hypoxic as reflected by an increase in lactate and alanine production paralleled by activation of hypoxia-inducible factor 1α (HIF1α). These changes indicate that anti-angiogenic treatment shifts energy production in glioma cells predominantly towards anaerobic glycolysis - a finding, which is further corroborated by the fact that glioma cells display decreased numbers of mitochondria upon BEV treatment. The metabolic switch towards anaerobic glycolysis is most likely due to changes in the tumor vasculature, since direct exposition of isolated glioma cells to BEV did not induce considerable changes in the metabolic profile [55]. Yet, these results have to be interpreted with caution, since only one human glioma cell line was used. The lack of detailed information about a metabolic switch in human tissue inspired us to address this question in human glioma cells and tissue samples together with an international consortium of neurooncological colleagues [56]. As previously reported in animal models, also human glioma cells displayed increased lactate production accompanied by reduced levels of metabolites necessary for the functioning of the tricarboxylic acid cycle. Along this line, expression levels of glycolytic enzymes were elevated upon BEV treatment suggesting a switch towards anaerobic glycolytic metabolism. Immunohistochemical analyses of post-BEV resection or autopsy samples revealed increased lactate dehydrogenase-A (LDH-A) expression not only in perinecrotic areas where LDH-A expression is commonly seen in treatment-naive samples but also in large vital tumor parts or even in single glioma cells diffusely infiltrating the surrounding CNS tissue. All these findings clearly point towards a metabolic adaptation process, which is not related to clonal selection of glioma cell subsets. Meanwhile, *in vivo* imaging techniques supporting an intratumoral metabolic switch upon anti-angiogenic treatment have been developed [57, 58].

In summary, there seems to be a discordant pattern: On the one hand, BEV treatment apparently enhances radiosensitivity by reducing tumor hypoxia during the vascular normalization phase, on the other hand a switch towards anaerobic glycolysis has convincingly been reported. It remains to be elucidated which of these mechanisms has higher relevance in clinical practice.

## THE CHANGING NEURORADIOLOGICAL FACE OF GLIOMAS UPON BEV-TREATMENT

One of the major reasons for the negative regulatory votum against BEV in the first line therapy of GBM is the relative uncertainty of imaging criteria during BEV treatment. The discrepancy between PFS prolongation and lack of OS benefit was attributed to the reduced permeability of the blood brain barrier (BBB) leading to a delayed detection of tumor recurrence – a phenomenon termed pseudo-response. Already in very early studies applying BEV in the context of recurrent gliomas, more than 75% of the patients showed at least a partial neuroradiologically confirmed treatment response [59]. Surprisingly, with 27 weeks the OS was only marginally longer than the PFS pointing towards disease stabilizing and/or progression masking effects rather than sustained long-term anti-tumor activity. A significant association of radiographic responses and high VEGF expression levels was observed, even though patient survival was not prolonged [60]. Pioneering neuroradiological work on BEV treatment in gliomas reported a significant reduction in the edema-to-tumor volume and a relative decrease in necrotic areas [61]. We could show that the contrast-enhancing tumor volume and edema declined significantly upon initiation of BEV treatment, whereas the non-contrast-enhancing tumor volume did initially not decrease but increased strikingly at progression [62]. However, these results should be interpreted with caution, since magnetic resonance (MR)-based differentiation between non-enhancing tumor volume and edema relies on signal intensities only. Other MR studies revealed that elementary MR parameters, such as the T1- and T2 relaxation times, can assess treatment response and also progressive tumor infiltration with higher sensitivity [58, 63, 64]. The relative cerebral blood volume (rCBV) has also been identified as an important neuroradiological parameter to predict the time to progression (TTP) in BEV-treated GBM patients [65]. In addition, we observed that T1-hyperintense lesions with diffusion restriction were positively associated with prolonged patient survival upon anti-angiogenic treatment [66, 67]. Parallel computed tomography (CT) imaging suggested the presence of calcifications in BEV-treated GBMs, which could be confirmed by means of histological analyses [67]. Meanwhile it became evident that more than 50% of all GBM patients treated with BEV develop similar imaging alterations, which seem to be predictors of an anti-angiogenic treatment response. Although attempts to categorize neuroradiological progression under anti-angiogenic treatment have been initiated, so far no official guidelines could be established [68].

In summary, the aforementioned novel neuroradiological findings, which are associated with anti-angiogenic therapy and have not been previously observed under standard radiochemotherapeutic regimens, fuel the still ongoing debate about pseudo-progression and/or pseudo-response [69]. This dilemma might best be resolved by detailed neuroimaging and neuropathological analyses in a setting allowing for kinetic correlation of MR changes and histopathology. Another option for the exclusion or confirmation of a pseudo-response after BEV treatment might be ^18^F-fluoroethyl-l-tyrosine positron emission tomography ([^18^F]FET-PET). Several reports have described obvious characteristics following BEV treatment, but so far no data on combined modality approaches are available [70-72].

## CURRENT STATUS OF IRRADIATION AND ANTI-ANGIOGENIC TREATMENT: WILL THE CARDS BE RESHUFFLED ?

The treatment of malignant brain tumors has been subject to a variety of clinical studies combining RT with BEV with or without TMZ [73]. In 2014, the results of two large randomized trials on BEV in primary GBM treatment have been published. In the AVAglio trial, combined radiochemotherapy with TMZ according to the Stupp regimen (EORTC 26981 - 22981 NCIC CE3 trial) [74, 75] was compared to the same regimen in combination with BEV. The median PFS in the BEV group exceeded that in the placebo group (10.6 months vs. 6.2 months; HR 0.64; 95% CI 0.55 to 0.74, p<0.001). Yet, the overall survival (OS) was not significantly different between both groups (HR 0.88; 95% CI 0.76 to 1.02, p=0.10), but improved maintenance of baseline quality of life and performance status were observed with BEV. Of note, the rate of adverse events was also higher in the BEV arm [33]. In the same issue of the New England Journal of Medicine, the results of the similarly designed RTOG 0825 trial were published. There was no significant difference in median OS between both treatment arms (15.7 or 16.1 months, respectively; HR for death in the BEV group 1.13). The median PFS was longer in the BEV group (10.7 months vs. 7.3 months; HR 0.79), but the pre¬¬specified significance level for PFS was not reached. In contrast to the AVAglio trial, where an improved quality of life was reported for the BEV group, the opposite if any change in the quality of life and a possible deterioration in neurocognitive performance was observed in the RTOG 0825 trial [34]. In summary, both phase III trials failed to show prolongation of OS by BEV-containing treatment regimens, whereas significant and marginally significant PFS benefits were described, and contradicting results were obtained when evaluating quality of life or neurocognition (no data within the AVAglio trial), respectively [33, 34].

The phase II GLARIUS trial explored the efficacy of BEV plus irinotecan compared to standard TMZ in the first-line radiochemotherapy of GBM patients with non-methylated O-6-methylguanine-DNA methyltransferase (MGMT) promoter. In the BEV/irinotecan arm, PFS was significantly prolonged from a median of 5.9 months (95% CI 2.7-6.2 months) to 9.7 months (95% CI 8.7-10.5 months, p=0.0004; HR 0.56, 95% CI 0.4-0.79). Nevertheless, no prolongation of the median OS and no improvement in the quality of life were observed [76]. In summary, two well-conducted randomized phase III studies and one phase II trial could not demonstrate significant OS benefits by adding BEV to standard radiochemotherapy for patients with newly diagnosed GBM.

One might come to discordant conclusions when considering the available clinical phase II/III results [33]. Apparently, there are improvements in terms of PFS, but these might derive from neuroradiological pseudo-responses. Moreover, the failure to show benefits in OS leaves little place for the notion of putative synergistic interactions between BEV and RT – at least in the context of radiochemotherapy. In contrast, the median OS in the BEV groups was clearly prolonged as compared to historical controls, and the substantial crossover, which has taken place within the trials and renders them statistically difficult to analyze, might account for the failure to reach the defined endpoint of OS prolongation. Therefore, longitudinal crossover correction analyses of the GLARIUS trial are currently in preparation, and the results are eagerly awaited. Given all these limitations, it currently remains unclear whether BEV administration in combination with radiochemotherapy can exert synergistic effects and clinical efficacy for the first line treatment of GBM patients. Clearly, treatment schedules as well as patient selection should be reassessed in order to derive more conclusive results in the future.

The decision of the EMA (European Medicines Agency) against BEV for the treatment of newly diagnosed GBM was based on the results of both RTOG 0825 and AVAglio. However, the observations of initial attempts to use BEV in recurrent glioma appear promising and inspired an ongoing second line chemotherapy trial (EORTC26101, BEV + lomustine), which might lead to the approval of BEV for the treatment of recurrent malignant glioma. This trial is based on the efficacy of BEV and lomustine within the BELOB phase II trial [77]. Accordingly, a reassessment in combination with RT might be possible in future.

## FUTURE DIRECTIONS

We have learned from the treatment of high grade gliomas with BEV in both preclinical models and clinical settings that anti-angiogenic therapies considerably change tumorbiological properties, including vascularization and tumor cell metabolism. These effects can be clearly demonstrated by means of neuroradiological and neuro¬pathological analyses. Nevertheless, although the morphological and physiological changes in the tumor were associated with prolonged PFS in several phase II/III trials the OS remained virtually unchanged. Which are the putative reasons for this and how should they be addressed in the future?

## CRITICAL REASSESSMENT OF THE VESSEL-NORMALIZATION CONCEPT AND ANTI-TUMORAL EFFECTS OF BEV IN THE CONTEXT OF COMBINATORY TREATMENT STRATEGIES

The concept of vascular normalization implies increased pericyte coverage as well as reduced basement membrane thickness of tumor vessels, and led to the hypothesis that these changes could favor tumor oxygenation, which in turn would improve treatment effects and thus patient survival [43, 78]. Already in the pioneering work of Winkler and colleagues the term “normalization window” was introduced reflecting that an optimal time window for RT in the context of anti-angiogenic treatment does exist [43]. However, it appears highly challenging to fine-tune the best moment of vascular normalization for combined treatment modalities in the clinical routine. From a neuropathological perspective, it is also difficult to understand how large glomeruloid vascular structures, once they have been established in the glioblastoma micromilieu, should undergo a normalization process, which could be exploited by radiochemotherapeutic approaches. Moreover, our intravital microscopy study revealed that regression of gliomas during anti-angiogenic therapy can occur independently of vascular normalization, suggesting that the underlying molecular mechanisms are multifactorial and comprise more aspects than vascular normalization only [48]. Hence, it still remains to be determined in detail if and by which means anti-angiogenic treatment can set a favorable ground for radio- and/or chemotherapy. This is of pivotal importance, since combined modality approaches might also have negative additive effects. For both irradiation and anti-angiogenic therapy, experimental studies have shown enhanced glioma cell invasiveness, and RT can also affect the tumor vasculature [79-82]. Accordingly, future research should aim at deciphering the putative positive synergistic effects of anti-angiogenic treatment and RT and particularly also their potentially detrimental consequences for GBM patients as well as the underlying mechanisms.

## WHO PROFITS - WHO SUFFERS: ATTEMPTS OF PATIENT STRATIFICATION

Despite encouraging results using anti-angiogenic therapy in GBM it appears that only a subset of patients experiences survival benefits when receiving BEV treatment, and striking differences in response rates and long-term tumor control have been observed. Biomarkers able to identify patients, who would specifically benefit from anti-angiogenic therapy in combination with radiochemotherapy still remain an unmet need not only in neurooncology [83, 84]. In this regard, baseline levels and/or variation of numerous intratumoral or circulating candidate biomarkers have been extensively explored. However, so far their predictive values remain weak, and they could rarely be confirmed among different studies [85]. A recent report suggested that circulating levels of VEGF-A are prognostic for the outcome of metastatic colorectal, lung and renal cell cancer, but they were not predictive for BEV-based treatment benefits [86]. Elevated VEGF-expression in recurrent malignant gliomas as determined by IHC, however, was associated with increased response rates to BEV but did not predict survival, whereas high expression of carbonic anhydrase 9 (CA9) was related to poor survival outcome [60]. Yet, in the AVAglio trial pretreatment plasma concentrations of VEGF and sVEGF did not show significant associations with PFS or OS, respectively [87]. In the context of BEV-treatment for recurrent GBM, increased baseline numbers of CD109^+^ circulating endothelial cells (CECs) identified a subgroup of patients with longer PFS and OS, which also encompassed more long-term responders [88]. Additionally, high MMP2 plasma levels were reported to be associated with treatment response and survival in patients with recurrent GBM under BEV treatment but not under cytotoxic chemotherapy alone [89]. Further research and randomized clinical trials are clearly needed in order to evaluate the predictive power of these potential biomarkers in the future.

Molecular genetic advances have contributed to a better understanding of GBM pathophysiology and might have the potential for disease stratification. The most recent, clinically relevant classification has been provided by The Cancer Genome Atlas consortium (TCGA). On the basis of gene expression profiling with respect to p53, epidermal growth factor (EGFR), neurofibromin (NFI), platelet-derived growth factor alpha (PDGFRA) and isocitrate dehydrogenase 1 (IDH1) four distinct molecular subtypes of GBMs (proneural, neural, classical, and mesenchymal) were defined [90]. Importantly, clear differences in prognosis were observed for these molecular subtypes with proneural GBM exhibiting relatively favorable prognosis – a finding that had already been reported before [90, 91]. Colman et al. further introduced a multigene predictor set of 9 genes associated with a mesenchymal (angiogenic) GBM phenotype and glioma stem cell markers, which appears to have considerable potential for optimizing GBM therapy and for identifying novel therapeutic approaches in order to specifically target GBMs that are refractory to standard therapy [92]. Hypothesizing that the marked differences in oncogenic and angiogenic drivers across distinct expression signatures might translate into differential responses to anti-VEGF therapy, patient stratification in the RTOG 0825 trial was dichotomized into favorable and unfavorable outcome based on this 9-gene panel. However, no prospectively defined subgroup of patients exhibited selective survival benefits from the early administration of BEV [34]. Preliminary molecular subgroup analyses on the basis of a 10-gene predictor of mesenchymal-subtype-associated genes warrants further testing [93]. Pilot data from the AVAglio trial suggest that the proneural molecular GBM subtype (tumors with IDH mutations excluded) might respond better to BEV treatment than the other three molecular subtypes [94].

In summary, for both phase III trials considerable efforts have been undertaken to define patient subgroups who would particularly profit from BEV treatment plus radiochemotherapy. Different strategies were followed. Whereas the RTOG 0825 consortium started with the Colman signature and subsequently defined a 9-/10-gene set of mesenchymal GBM [92], the AVAglio team employed subtyping according to Philipps and Verhaak and could show that for some GBM subgroups BEV treatment was effective, while for others detrimental effects could be observed [90, 94]. So far, these subgroup analyses remain on an exploratory level, and the predictive significance of the molecular genetic signatures has to validated. Consequently, independent evaluation is planned within the GLARIUS cohort, and the results are awaited with great interest. Taken together, to date no GBM subtype has been identified to be “advantageous” when using radiochemotherapy plus BEV, and currently no reliable a priori evaluation does exist in order to determine whether or not a patient will profit from BEV therapy. Independent cross-trial confirmation of putative predictive biomarkers is lacking, and so their use in current clinical practice has to be discouraged [41]. Further efforts are required in order to examine the value of TCGA-based transcriptional classification as well as other putative biomarkers with the aim of increasing the likelihood of successful of anti-angiogenic treatment.

## IRRADIATION AND ANTI-ANGIOGENIC TREATMENT: BROTHERS IN ARMS OR UNHOLY ALLIANCE?

The interaction between VEGF signaling and RT has been addressed in several preclinical studies [43, 95-100]. Irradiation reportedly stimulates the upregulation of VEGF in different glioma cell lines and xenografts [17, 98], and interfering with VEGF signaling by neutralizing antibodies has been shown to enhance the anti-tumor effects of ionizing radiation more than additively [43, 95, 101]. Notably, scavenging VEGF with BEV increased the sensitivity of both tumor and endothelial cells to the cytotoxic effects of ionizing irradiation. Hence, improved tumor control in glioma xenografts by combined administration of BEV and RT was attributed to the abrogation of irradiation-induced VEGF signaling and better tumor oxygenation due to vascular normalization [11, 43].

The clinical efficacy of BEV application in combination with RT as assessed by PFS and OS is currently being extensively discussed. However, one aspect remains commonly disregarded in this context: The beneficial impact of BEV on attenuating radiation-induced cerebral necrosis. To our knowledge, there has been only one small prospective randomized trial addressing this issue, and it provided clear evidence for the successful treatment of radiogenic brain necrosis with BEV [102]. This is of particular interest, since dose escalation has been a relevant topic in the field of RT for GBM over years. It has never definitely been proven that a dose-response-relationship above 60 Gy does exist, and there are different reasons why several clinical trials failed [103-107]. Yet, the high frequency of local and/or in-field GBM recurrence renders insufficient local tumor control the most plausible explanation [108-110]. Tsien et al. showed in a phase I trial that gradual dose escalation up to 84 Gy is feasible with an acceptable risk of late central nervous system toxicity. Unfortunately, no data on concomitant TMZ administration are available, and overall patient numbers were small [111]. BEV treatment could be a valuable strategy in the context of dose escalation, since the induction of radiation necrosis in the brain is a major concern in this regard [112]. Administration of BEV could be utilized in order to counteract the onset and/or aggravation of radiogenic brain necrosis, and irradiation doses could be increased with the aim of improving local tumor control. The reasons why BEV can prevent radiation necrosis are poorly understood. Hypotheses range from a reduction in interstitial edema, a decrease in interstitial hypertension to a reduction in the permeability of leaky vasculature, and others. Notably, some of these also are supposed to underlie the wanted synergistic interactions with RT in terms of tumor control [18]. Individual case reports underscore the dramatic effects of BEV when treating symptomatic, radiation-induced cerebral necrosis [113, 114]. Hence, it might be worth to consider increasing the irradiation dose to achieve improved tumor control and administering BEV for the protection of the normal brain parenchyma – at least on a very careful and case-by-case basis. In such aggressive treatment regimens, the potentially negative influences on neurocognition should always be payed attention to and need to be properly assessed.

The increased risk of radiation necrosis was an initial concern when the concept of re-irradiation of malignant glioma emerged. However, in recent years this treatment option has been adopted as safe and effective [115-121]. For recurrent malignant glioma, BEV was repeatedly used in combination with re-irradiation. One group tested the sequential administration of radiosurgery and BEV with favorable outcome [122]. Complementarily, Gutin and co-workers determined the safety and activity of RT and concomitant BEV treatment. Here, PFS after 6 months (PFS-6) reached 65% for the GBM cohort [14]. In a previous retrospective study on 30 patients (20 being treated with BEV and 10 without BEV), we could show that PFS-6 within the BEV-treated group was 72%, and OS was significantly prolonged [123]. In a second study with substantially longer follow-up and a higher patient numbers, the significant post-recurrence survival (PRS) benefit obtained by BEV application could be confirmed, and a low incidence of side-effects was observed [124]. Along these lines, there is an ongoing RTOG 1205 trial, which aims at evaluating improvements in OS for patients with recurrent GBM receiving BEV and re-irradiation as compared to patients receiving BEV alone. Other trials employing BEV and re-irradiation also provided favorable results [125-127].

One further concern about anti-angiogenic treatment in the context of RT is an increased stimulation of glioma cell migration and invasiveness. Results from preclinical studies have shown that (perivascular) invasion increases during anti-VEGF therapy and might thereby limit therapeutic efficacy [50, 128-130]. Tumor cells within the invasive margins of GBMs can escape anti-angiogenic therapy and local irradiation, because they can migrate into surrounding areas of normal brain parenchyma, which are located outside the irradiation field and whose intact blood-brain barrier prevents BEV from entering the tissue. However, these tumor cells have been predicted to be susceptible to irradiation-induced abrogation of clonogenic survival providing a further rationale for combining anti-angiogenic therapy with irradiation of the tumor surrounding margins, yet at lower doses [99, 131]. Initial clinical reports revealed that anti-VEGF therapies were associated with non-contrast-enhancing radiographic tumor progression, which was interpreted as an increase in tumor invasiveness [35]. Available clinical data, however, are very heterogeneous, and the major studies do not uniformly support the assumption that BEV treatment induces invasive growth at time of recurrence [33, 36, 132, 133]. If a more invasive GBM phenotype emerged during therapy, this should become obvious in a higher frequency of out-of-field recurrences due to increased tumor cell migration [134]. Although a numerically slightly higher rate of diffuse progressions was observed in the BEV arm of the AVAglio study, this did not significantly affect OS. However, the initial growth pattern (diffuse vs. non-diffuse) was per se prognostic. In patients who had non-diffuse tumors at baseline, median OS was 20.1 (BEV arm) and 18.4 months (standard arm), whereas for tumors with diffuse growth patterns at baseline, median OS was 15.6 (BEV arm) and 16.2 months (standard arm), respectively [38]. When examining the relapse patterns in patients with recurrent malignant glioma under BEV treatment in combination with re-irradiation, we observed mainly centrally located recurrences [135]. A second argument commonly listed when discussing the potential stimulation of glioma invasiveness under BEV treatment is that in view of improved PFS and equal OS in the BEV arms of AVAglio and RTOG 0825, post-PFS survival apparently is decreased in comparison to non-BEV treated patients. Yet, this issue is difficult to evaluate, since the relevant crossover rates within both trials render them statistically complex to analyze (31% within the AVAglio trial, RTOG 0825 even higher). Crossover correction analyses are required in order to address this concern comprehensively.

Finally, a major caveat of BEV application in combination with RT in GBM remains: detrimental neurocognitive effects. Unfortunately, RTOG 0825 and AVAglio, do not provide conclusive results in this regard. From a physiological point of view, this hypothesis cannot be declined, since VEGF is a relevant neuroprotector and its targeting might give rise to possible negative side effects of RT. Available literature on this topic delineates a similar picture in animal models, where VEGF apart from stimulating microvascular proliferation and angiogenesis - enhances neuronal differentiation, protection, and regeneration [39, 136]. Therefore, it is conceivable to assume that VEGF blockade augments irradiation-induced neurological toxicity [137, 138]. Concerning neurocognitive performance, the RTOG 0825 consortium, in contrast to the AVAglio consortium, incorporated formal neurocognitive testing in order to assess cognitive performance. Intriguingly, adverse effects on processing speed and executive functions in patients receiving BEV as compared to patients in the standard arm were noted [34].

## CONCLUSIONS

Overall, it remains unclear how the therapeutic alliance of RT and BEV application for GBM will develop. In our opinion, there are several open questions, which need to be addressed in the future.

Are there subgroups of patients who are specifically eligible or not suitable for BEV/RT treatment?

Having predictive biomarkers would be highly advisable, and a validation of already defined subgroups is of utmost importance.

What are the molecular mechanisms of BEV-mediated radioprotection of normal brain parenchyma and might dose-escalation make sense when detrimental effects on the normal tissue can be avoided?

It is relevant to delineate in how far dose escalation with concomitant BEV treatment is feasible.

After RT/BEV treatment, how long should maintenance BEV therapy be performed?

Some of the adverse effects could be related to sustained BEV treatment alone and not to the combined RT/BEV regimen.

**Figure 1:**
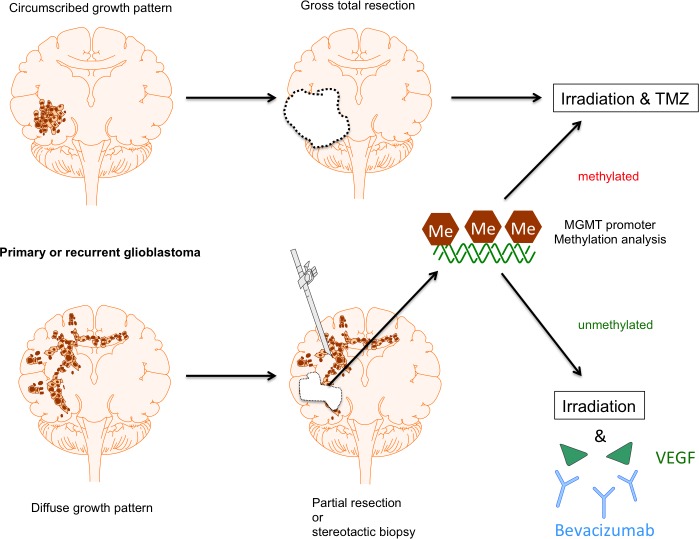
Proposal of patient stratification for BEV treatment in GBM For GBM patients with gross total resection standard treatment including radiochemotherapy with TMZ is still considered to be the most promising therapy. The same applies for both completely and partially resected GBMs with MGMT promoter methylation, since this subcohort shows a significant benefit from TMZ chemotherapy. In contrast, patients with incompletely resected GBM in conjunction with a non-methylated MGMT promoter might considerably profit from (hypo)fractionated (re-)irradiation in combination with BEV treatment.

Clearly, future trials need to focus on patient subgroups for whom real benefits in terms of PFS and OS can be expected as depicted in Figure 1. Patients with both primary GBM or recurrent malignant glioma (pre-irradiated) should be considered. Out of these, patients with gross total resection should be excluded, since tortuous vessel formations, for which the combined effects of BEV and RT might be particularly helpful, are unlikely to be present. The same would apply to GBMs with methylated MGMT promoter, since RT plus TMZ alone is the treatment of choice in these cases, and there is little probability that BEV can add relevant improvement after two phase III trials without OS benefits. If a certain molecular subgroup, such as the mesenchymal GBM type, should be excluded continues to be unclear, and further studies addressing this question are needed. The remaining tumors will be the inoperable ones or the ones where only subtotal resection is possible due to the involvement of critical CNS structures. In these cases, dose escalation with concomitant BEV treatment might be considered and (hypo)fractionated re-irradiation might be a therapeutic option. The optimal duration of subsequent maintenance therapy with BEV remains yet to be determined, since it has not been proven that a perpetuation exerts relevant benefits and potential adverse side effects might be caused by long-term BEV treatment per se [139].

One facet of BEV treatment, which is currently moving into the focus of interest, and has not been intensively discussed here, are the immunological consequences of anti-angiogenic therapy. The tumor microenvironment and particularly the tumor endothelium appear to strongly impact on the regulation immune mechanisms and thus contribute to the establishment of an immunosuppressive milieu. VEGF has been attributed an essential role in this regard. Accordingly, interfering with VEGF function is known to have local as well as systemic immunological effects, which - not only due to the advent of immunotherapeutic approaches - certainly will be further examined in future studies [140, 141].
